# Lossy Mode Resonance Sensors Based on Anisotropic Few-Layer Black Phosphorus

**DOI:** 10.3390/nano14090736

**Published:** 2024-04-23

**Authors:** Yanting Shen, Qifeng Zhu, Zhuo Chen, Jiawei Wu, Binghuang Chen, Enwen Dai, Weiqing Pan

**Affiliations:** School of Science, Zhejiang University of Science and Technology, Hangzhou 310023, China

**Keywords:** lossy mode resonance, few-layer black phosphorus, anisotropy, sensing performance

## Abstract

Lossy mode resonance (LMR) sensors offer a promising avenue to surpass the constraints of conventional surface plasmon resonance (SPR) sensors by delivering enhanced label-free detection capabilities. A notable edge of LMR over SPR is its excitation potential by both transverse electric (TE) and transverse magnetic (TM) polarized light. Yet this merit remains underexplored due to challenges to achieving high sensing performance under both TM and TE polarization within a singular LMR model. This study introduces a theoretical model for an LMR prism refractive index sensor based on a MgF_2_-few layer black phosphorus-MgF_2_ configuration, which can achieve angular sensitivity nearing 90° refractive index unit^−1^ (RIU^−1^) for both polarizations. Leveraging the distinct anisotropic nature of black phosphorus, the figure of merit (FOM) values along its two principal crystal axes (zigzag and armchair) show great difference, achieving an impressive FOM of 1.178 × 10^6^ RIU^−1^ along the zigzag direction under TE polarized light and 1.231 × 10^4^ RIU^−1^ along the armchair direction under TM polarized light. We also provide an analysis of the electric field distribution for each configuration at its respective resonant conditions. The proposed structure paves the way for innovative applications of anisotropic-material-based LMR sensors in various applications.

## 1. Introduction

Lossy mode resonance (LMR) is a distinctive optical resonance phenomenon that occurs when waveguide modes couple with guided modes characterized by a complex effective index, known as lossy modes. This intriguing phenomenon, first discovered in 1993 [1], exhibits a remarkable sensitivity to environmental fluctuations. Similar to surface plasmon resonance (SPR), the resonance angle of LMR exhibits significant variations when there is a change in the refractive index of the sensing medium, which highlights the high angular sensitivities of LMR. As such, LMR has emerged as a pivotal detection signal in physical, chemical, and biological sensors for refractive index detection [2], voltage measurement [3], pH evaluation [4], and organic vapor inspection [5].

Compared to traditional SPR, which relies on the excitation of collective oscillations of electrons at metal–dielectric interfaces, LMR offers distinct advantages. Firstly, SPR modes can couple with both transverse electric (TE) and transverse magnetic (TM) polarized light in nanostructures, such as nanospheres in chains [6], nanowaveguides [7], nanotips [8], and so on. However, they cannot couple with TE polarized light in classical macroscopic structures. In contrast, LMR can be excited by both TE and TM polarized light but is not limited to nanoscale structures. Secondly, SPR is limited to metals, commonly gold and silver, while LMR can be realized in many types of excitation materials, including dielectric, transparent conducting oxides (TCOs), and polymers [9], broadening the range of design possibilities and applications. Therefore, LMR can provide higher quality factors and improved field confinement compared to SPR, leading to enhanced sensitivity and detection capabilities. Consequently, LMR sensors hold the potential to transcend SPR sensor limitations by offering superior sensing performance. In particular, the figure of merit (FOM) of LMR sensors is usually much higher than that of SPR sensors, which is one of the most important advantage that distinguishes them from SPR sensors.

Del Villar et al. in 2010 were the first to demonstrate the LMR phenomena by coating indium tin oxide (ITO) on optical fibers. In 2015, they further observed both SPR and LMR phenomena by using a Kretschmann configuration via a BK7 glass prism with all sides polished and coated with ITO [10,11,12], which paved the way for the investigation of LMR sensors based on the Kretschmann configuration. Typically, these configurations comprise three layers: a matching layer, a lossy layer, and a sensing layer. The matching layer is sandwiched between the prism and the lossy layer. The refractive index and thickness of the matching layer influences the light coupling between the evanescent wave and the lossy mode in the lossy layer, which could improve the curve of resonance dip and field distribution. As the Kretschmann configuration is based on the principle of attenuated total reflection (ATR), the refractive index of the matching layer film should be lower than the refractive index of the prism. The LMR can be strongly excited when light penetrates the matching layer and is trapped by the lossy layer under a specific matching condition [13,14]. And the lossy layer serves as the LMR excitation medium. Del Villar et al. summarized the LMR excitation conditions in 2017, emphasizing the refractive index properties of the lossy layer. Specifically, the real part $\epsilon_{r2}$ of the refractive index of the lossy layer is positive and is large enough (larger than the real part $\epsilon_{r3}$ of the refractive index of the sensing layer), and the imaginary part $\epsilon_{i2}$ is small enough [15]. However, a majority of existing LMR sensors struggle to excite both TM and TE waves with high sensitivity within a singular structure [13].

To enhance LMR sensor performance, researchers have explored various 2D materials with appropriate refractive indices for the lossy layer [16,17,18]. In 2018, graphene was introduced as a lossy material and achieved a figure of merit (FOM) of 410 refractive index unit^−1^ (RIU^−1^) [19]. Subsequently, there has been a surge in the integration of two-dimensional materials into LMR sensors. Notable studies include the application of transition metal dichalcogenides (TMDs) for high sensitivity and FOM detection [20,21]. In 2021, an LMR structure based on the perovskite nanomaterial CH_3_NH_3_PbBr_3_ was proposed and boasted intensity sensitivities of 11,382 RIU^−1^ under TM incident light and 21,697 RIU^−1^ for TE light [22].

Black phosphorus (BP), a second-generation graphene-like 2D material, exhibits excellent photoelectric performance [23,24] and mechanical properties [25,26] and high carrier mobility [27]. Compared to other 2D materials, e.g., transition metal dichalcogenides (TMDs), its relatively large real refractive index component, low imaginary part, and reduced out-of-plane electrical conductance make it an ideal candidate for the LMR lossy layer. Wu et al. proposed a high-performance LMR sensor based on few-layer BP (FLBP) with a high FOM of 2 × 10^5^ RIU^−1^ for TM polarized light [28]. In 2022, Zhang et al. introduced an LMR structure similar to the SPR Otto configuration, employing FLBP as the lossy layer. By leveraging the sensing material as the matching layer, this sensor’s performance was significantly augmented, achieving resonant angular sensitivities between 83–420° RIU^−1^ under TE light and an FOM of $9.9\times 10^{5}$ RIU^−1^ for TM light [13].

One of the most significant properties of FLBP is its distinct anisotropy in phonons, photons, and electrons [29,30,31,32]. Incident polarized light divides into x- and z-polarized components along the zigzag (zz) and armchair (ac) principal crystal axes of FLBP and moves at varying velocities [33]. Consequently, considering the twist angle of each BP film layer, the refractive index of FLBP films under different stacking modes exhibit significant variations [34,35,36]. Yet, prior studies on FLBP-based LMR sensors have scarcely addressed the implications of these anisotropic properties.

In this paper, we propose a theoretical model of an LMR prism refractive index sensor based on a MgF_2_-FLBP-MgF_2_ configuration. By leveraging the anisotropic nature of FLBP, we modulate the sensing performance along its two principal crystal axes (zz and ac) under TE and TM polarized illumination. Our findings underscore the potential of this LMR model to achieve remarkable angular sensitivity of 89.76° RIU^−1^ for both polarizations and FOM values of 1.178 × 10^6^ RIU^−1^ under TE polarized light and 1.231 × 10^4^ RIU^−1^ for TM polarized light. Furthermore, the mechanism of the resonance variation is examined by electric field distribution calculation. We can achieve high sensing performance under both polarizations within a singular sensor configuration by simply rotating it 90°, which is essentially rotation of the crystallographic axes of FLBP. We believe the proposed MgF_2_-FLBP-MgF_2_ structure will provide valuable design ideas for future anisotropic-material-based LMR sensors and find promising application in chemical and biological signal detection.

## 2. Materials and Methods

**Figure 1:**
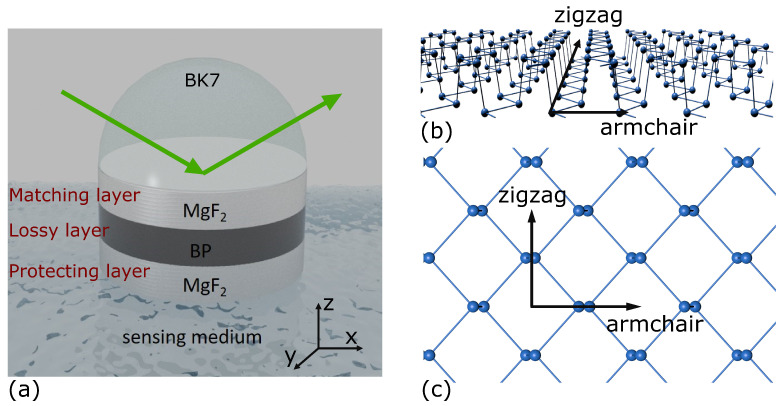
(**a**) The LMR configuration based on a MgF_2_-FLBP-MgF_2_ structure: (**b**,**c**) are the side view and the top view of the monolayer BP, respectively, and illustrate the zigzag (zz) and armchair (ac) principal crystal axes.

The LMR structure employed in this study adopts the Kretschmann configuration, featuring a sandwiched MgF_2_-FLBP-MgF_2_ structure to excite LMR, as depicted in Figure 1a. According to the band gap of FLBP, the excitation wavelength should be in the visible wavelength range. In our study, we have chosen to use a 532 nm laser as the incident light. This wavelength falls within the high absorption range of both BP and biological cells, making it suitable for our FLBP-based LMR excitation. This incident light traverses free space to reach a BK7 prism hemisphere ($n_{p}$ = 1.5195) under angular interrogation [37]. In the context of biosensing applications, the refractive index of the sensing medium is approximately 1.38 [13,38,39]. In order to optimize the sensing performance to its fullest potential, the refractive index of the matching layer is ideally matched to the refractive index of the sensing medium, which is 1.38. The upper ${\mathrm{MgF}}_{2}$ layer ($n_{1}$ = 1.38) serves as the matching layer and amplifies the LMR signal and adjusts the resonant angle. FLBP functions as the lossy layer. Due the rapid oxidation rate of BP, an additional ${\mathrm{MgF}}_{2}$ layer is affixed at the bottom to act as an oxygen barrier and provide anti-corrosion protection.

As a puckered anisotropic 2D material, FLBP’s refractive index is significantly influenced by its thickness and stacking sequences. This, in turn, impacts the sensitivity and FOM of LMR sensors. Consequently, optimizing the stacking sequences of FLBP and the thicknesses of both the matching and lossy layers is crucial. The monolayer BP plane features two non-equivalent directions: armchair (ac) and zigzag (zz), as illustrated in Figure 1b,c. Drawing from prior experimental studies and the Cauchy absorption model [40,41,42,43,44,45], FLBP exhibits refractive indexes of $n_{\mathrm{zz}}=3.56\;+\;0.126i$ in the zz direction and $n_{\mathrm{ac}}=3.29\;+\;0.428i$ in the ac direction, which indicates significantly lower imaginary parts of the refractive index compared to bulk BP. Compared to typical metal oxides, e.g., TiO_2_ ($n_{TiO_{2}}$ = 1.977 + 0.05i) and ZnO ($n_{ZnO}$ = 1.71749 + 0.066i), though, the imaginary part of the refractive index of BP is not relatively small: the real part is larger. From previous studies on LMR sensors based on metal oxides and BP [13,14,46], we can expect the BP structures to exhibit better sensing performance.

To analyze the multilayer structure stacked along the Z-axis, we employ the transfer matrix method:

(1) $$\left[\begin{array}{c} E_{1}\\ H_{1} \end{array}\right]=M\left[\begin{array}{c} E_{n-1}\\ H_{n-1} \end{array}\right],$$

where $E_{1}$ and $H_{1}$ represent the electric and magnetic fields, respectively, at the first layer’s boundary.

These fields correlate with the tangential electric field $E_{N-1}$ and magnetic field $H_{n-1}$ at the boundary of the N*^th^* layer through a characteristic matrix M:

(2) $$M=\prod_{j=2}^{N-1}M_{j}=\left[\begin{array}{cc} M_{11} & M_{12}\\ M_{21} & M_{22} \end{array}\right],$$

with

(3) $$M_{j}=\left[\begin{array}{cc} cos\beta_{j} & -\frac{i}{q_{j}}sin\beta_{j}\\ -iq_{j}sin\beta_{j} & cos\beta_{j} \end{array}\right].$$

For TM polarized light, $q_{j}={\left(\varepsilon_{j}-n_{1}^{2}\cdot sin^{2}\theta \right)}^{1/2}/\varepsilon_{j}$. For TE polarized light, $q_{j}={\left(\varepsilon_{j}-n_{1}^{2}\cdot sin^{2}\theta \right)}^{1/2}$. For both polarized lights, $\beta_{j}=2\pi d_{j}{\left(\varepsilon_{j}-n_{1}^{2}\cdot sin^{2}\theta \right)}^{1/2}/\lambda$, where $d_{j}$ denotes the thickness of each layer, λ is the incident light’s wavelength, and θ represents the incident angle.

The reflection coefficient can be expressed as:

(4) $$r=\frac{\left(M_{11}+M_{12}q_{N}\right)q_{1}-\left(M_{21}+M_{22}q_{N}\right)}{\left(M_{11}+M_{12}q_{N}\right)q_{1}+\left(M_{21}+M_{22}q_{N}\right)}.$$

The light intensity of the multilayer structure is normalized, yielding the reflectivity:

(5) $$R=|r{|}^{2}.$$

A sensor’s sensitivity is typically characterized in three ways: intensity sensitivity, phase sensitivity, and resonant angular sensitivity. In this study, we primarily focus on resonant angular sensitivity for the proposed LMR sensor, which gauges the resonant angular shift due to the sensing layer’s refractive index change (Δn). This can be articulated as:

(6) $$S=\frac{\Delta \theta }{\Delta n}.$$

The quality factor is defined as:

(7) $$FOM=S\cdot \frac{1}{FWHM}.$$

The refractive index variation of the sensing medium is set as Δn=0.0002; Δθ indicates the resonance angle variation with different sensing media. The finite element analysis (FEA) method facilitates the electric distribution calculations. Floquet periodic boundary conditions are implemented, and excitation is introduced through a periodic input port on the surface of the prism. The modified electronic band structure of FLBP obtained from DFT calculations is incorporating into our calculation [47,48]. Derived from FEM, the simulation results are influenced by the mesh size. Therefore, in order to accurately model the LMR electric distribution in our structure under the potential impact of nanoscale confinement, the mesh size is refined, and thus, the results converged. We use the physics-controlled mesh and set the maximum mesh element size to be 1/M times the thickness of monolayer BP ($d_{1LBP}/M$). By carefully controlling the value of M, the mesh refinement can be sufficiently stable and can meet the accuracy requirements.

## 3. Results and Discussion

In our proposed MgF_2_-FLBP-MgF_2_ LMR structure, we analyzed both TE and TM incident conditions. For TE waves, we first examined the variations in sensing parameters induced by different BP crystallographic axes (zz and ac) relative to the incident plane. The optimized thickness of the first layer (matching layer) ${\mathrm{MgF}}_{2}$ is $d_{1}=2000\;$ nm, and that of the third layer (protective layer) ${\mathrm{MgF}}_{2}$ is $d_{3}=100\;$ nm. The second layer (lossy layer) comprises a monolayer BP (1LBP) with a thickness of $d_{2}=0.53\;$ nm, which yields better sensing performance than other FLBPs. Detailed comparison are demonstrated in Figures S2 and S4. The refractive index of the sensing medium is varied from $n_{s}=1.38$ to 1.3808 in increments of 0.0002. By maintaining the thickness of the lossy layer constant, we can manipulate the sensing performance by alternating between the zz and ac crystallographic directions, which is achievable by simply rotating the sensor by 90°.

**Figure 2:**
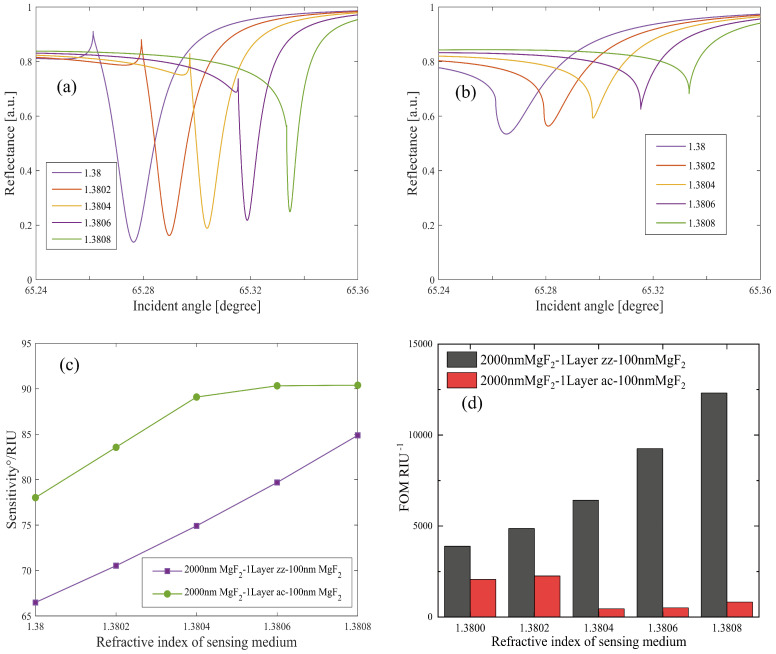
Comparison of the LMR sensing performance of the “2000 nm MgF_2_-1L zz BP-100 nm MgF_2_” structure and the “2000 nm MgF_2_-1L ac BP-100 nm MgF_2_” structure under TE polarized light: (**a**,**b**) the reflectance of structures with monolayer zz BP and monolayer ac BP, respectively; (**c**) the resonant angular sensitivity and (**d**) FOM of the two structures.

Figure 2a,b present the reflectivity of the MgF_2_-1LBP-MgF_2_ structure with the monolayer BP in the zz and ac directions, respectively. The performance disparities between the two structures are evident. The LMR dip of the zz BP structure is deeper than that of the ac BP structure, and its FWHM is narrower. Conversely, the ac BP structure exhibits a broader angular distribution with varying $n_{s}$. As a result, the ac BP structure boasts a higher angular sensitivity of 89.76° RIU^−1^, as illustrated in Figure 2c. Figure 2d highlights the FOMs of both structures. Clearly, the zz BP LMR sensor’s FOM is superior, especially with increasing $n_{s}$, which is attributed to the electronic states’ energy confinement in zz stacking BP arising from stronger interlayer interactions between adjacent layers [24,47]. The maximum FOM reaches 1.231 × 10^4^ RIU^−1^. The FOM results also suggest that FWHM exerts a more significant influence on FOM than sensitivity does. Comparing the zz and ac BP structures underscores the pivotal role of FLBP’s crystalline directions in determining LMR sensor performance.

**Figure 3:**
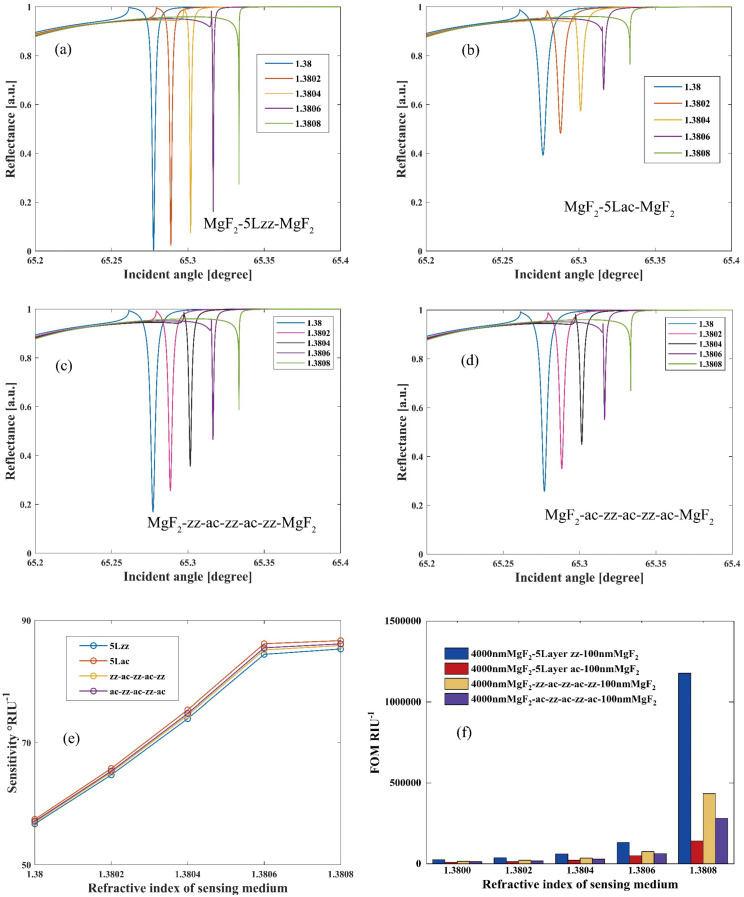
Reflectivity of “MgF_2_-5LBP-MgF_2_” LMR sensors under TM light with (**a**) 5zz stacking BP, (**b**) 5ac stacking BP, (**c**) zz-ac-zz-ac-zz stacking BP, and (**d**) ac-zz-ac-zz-ac stacking BP. (**e**) Sensitivity and (**f**) FOM of “MgF_2_-5LBP-MgF_2_” LMR sensors under TM light with different BP stacking sequences.

Additionally, we analyze the ${\mathrm{MgF}}_{2}$-FLBP-MgF_2_ LMR structure under TM polarized light, as illustrated in Figure 3. For TM illumination, the ideal thickness for the matching layer, lossy layer, and protective layer are determined to be $d_{1}=4000$ nm, $d_{2}=2.65$ nm (5-layer BP), and $d_{3}=100$ nm, respectively. For a five-layer BP structure, the interlayer distance exhibits a difference of approximately 0.0895 nm between the zz-zz/ac-ac stacking and zz-ac/ac-zz stacking configurations [49]. However, this discrepancy is relatively small and can be considered negligible. Therefore, it is reasonable to keep the thickness of a five-layer BP structure with different stacking sequences at a constant value of 2.65 nm. Detailed optimization analyses are presented in Figures S3 and S5. Figure 3 displays the sensing performance of MgF_2_-5LBP-MgF_2_ sensors with four different BP stacking sequences, including 5zz, zz+ac+zz+ac+zz, ac+zz+ac+zz+ac, and 5ac. Remarkably, for the zz BP structure, at $n_{s}=1.38$, the coupling of the lossy mode and waveguide mode peaks at 65.28°, which is attributable to the radiation field being confined within the lossy layer, resulting in robust LMR excitation. This leads to the relative absorption reaching 1, while the reflective power approaches 0. This finding suggests that modifying the BP crystallographic direction can significantly enhance the reflectance curve of the LMR sensors. Furthermore, the LMR resonant angle distributions for all four stacking sequences are strikingly similar, yielding closely matched sensitivities. However, due to the zz structure’s significantly narrower FWHM, its FOM is substantially higher, as indicated in Figure 3f. Specifically, at $n_{s}=1.3808$, the FOM can reach 1.178 × 10^6^ RIU^−1^. These analyses under both TE and TM polarizations demonstrate that exceptional sensing performance can be achieved by adjusting the crystallographic directions of FLBP under different polarization conditions.

The sensing performance of the BK7-50 nm Au SPR sensor, which utilizes a similar Kretschmann configuration, has also been analyzed and is illustrated in the Supplementary Material in Figure S1. This sensor exhibits a significantly higher angular sensitivity, nearly 200° RIU^−1^, compared to the 90° RIU^−1^ of the BP LMR sensor. However, its FOM is only 34.6 RIU^−1^, which is markedly lower compared to the impressive $1.178\times 10^{6}$ RIU^−1^ FOM of the BP LMR sensor. This comparison clearly demonstrates the primary advantage of LMR sensors over SPR sensors: their substantially higher FOM.

**Figure 4:**
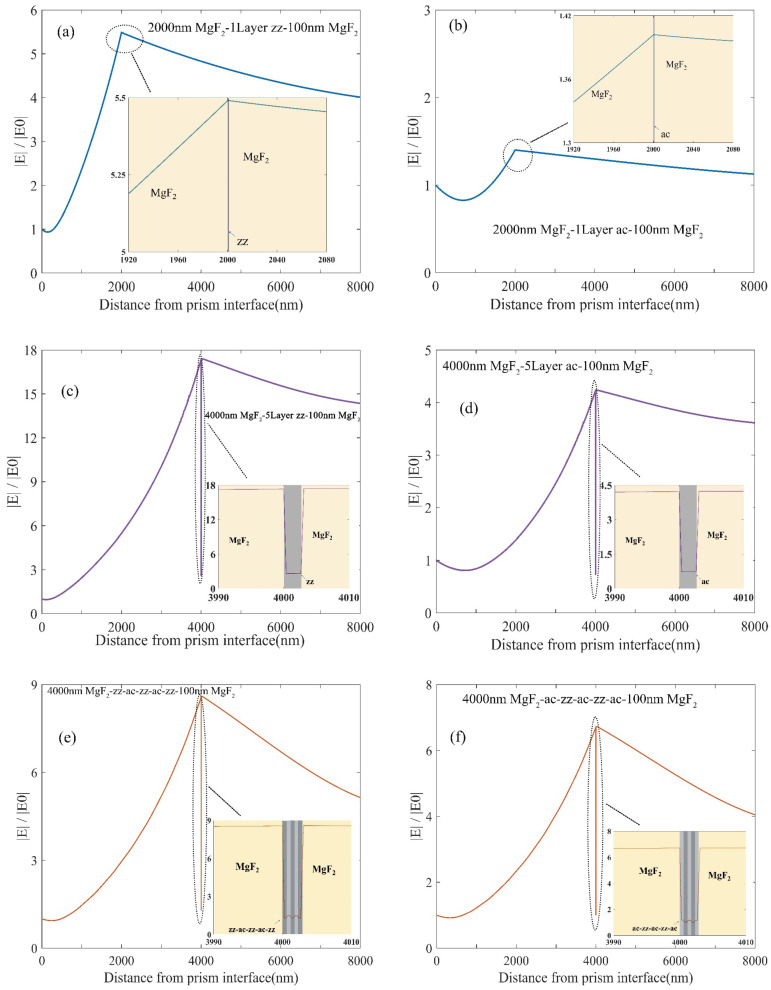
The electric field distribution of the proposed structures: (**a**) 2000 nm MgF_2_-1L zz BP-100 nm MgF_2_; (**b**) 2000 nm MgF_2_-1L ac BP-100 nm MgF_2_; (**c**) 4000 nm MgF_2_-5L zz BP-100 nm MgF_2_; (**d**) 4000 nm MgF_2_-5L ac BP-100 nm MgF_2_; (**e**) 4000 nm MgF_2_-zz+ac+zz+ac+zz BP-100 nm MgF_2_; (**f**) 4000 nm MgF_2_-ac+zz+ac+zz+ac BP-100 nm MgF_2_. The embedded diagrams of each figure are the magnified electric field distribution around the FLBP interfaces.

To gain deeper insights into the distinct sensing performances of the proposed sensor structures, we employ finite element analysis to explore the electric field distribution of each structure under its specific resonant conditions. Figure 4a,b depict the electric field distribution of the 2000 nm MgF_2_-1LBP-100 nm MgF_2_ structure under TE polarization with one-layer zz and ac BP, respectively. This reveals that the LMR resonance with the ac BP structure is considerably lower than that with the zz BP structure. Figure 4c,f display the electric field distribution of the 4000 nm MgF_2_-5LBP-100 nm MgF_2_ structure under TM polarization with four types stacking BP. All four structures exhibit increasing electric fields, but the difference in the electric field at the zz and ac interfaces is pronounced. The maximum electric field at the zz BP interface exceeds that at the ac BP interface by over fourfold. The electric fields at the zz+ac+zz+ac+zz and ac+zz+ac+zz+ac interfaces are in between the fields of five-layer zz and ac structures, aligning with the aforementioned sensitivity and FOM analysis. From previous research [50], it is crucial to consider nanoscale quantum corrections in the dielectric functions of all materials within the structure when solving the Maxwell–Fresnel problem using the FEA method. Neglecting these corrections may lead to inaccuracies. Although the refractive indices of the FLBPs used in our model are derived from a modified FLBP electronic band structure obtained through DFT calculations and experiments, the absolute value of our electric field distribution might not be accurate, while the comparison analysis of the electric field distribution of LMR sensors with different FLBP stacking sequences still holds significant value. For future work, we recommend adjusting the dielectric function of FLBP by incorporating modified photon absorption parameters to further enhance the accuracy of our calculations.

## 4. Conclusions

In summary, this paper introduced a theoretical model of an LMR sensor based on the MgF_2_-FLBP-MgF_2_ structure, employing ${\mathrm{MgF}}_{2}$ as the matching layer and FLBP as the lossy layer. Both TE and TM polarized light can excite the LMR signal. The thickness of each layer is optimized. We conducted an in-depth analysis of FLBP’s anisotropy and its impact on sensing performance. Under TM polarization, a maximum sensitivity of 89.76° RIU^−1^ is achieved with monolayer BP in the ac direction, while the highest FOM of 1.231 × 10^4^ RIU^−1^ is obtained with monolayer BP in the zz direction. Under TE polarization, the maximum sensitivity is 86.68° RIU^−1^, with the highest FOM reaching an impressive 1.178 × 10^6^ RIU^−1^ with five-layer BP in the zz direction. By adjusting the crystallographic direction of the BP layer, which can be practically achieved by simply rotating the Kretschmann configuration, the sensor’s performance under both TE and TM polarized light can be modulated. Our findings underscore the importance of the crystallographic direction of FLBP and offer a fresh perspective on harnessing the anisotropic properties of materials for LMR sensor applications. For future works, a comprehensive exploration into the sensing performance of LMR sensors integrated with 2D materials with varied twist angles or stacking sequences could unveil innovative strategies for precision-tuned, high-performance sensing applications.

## Data Availability

The data presented in this study are available upon reasonable request from the corresponding author.

## Supplementary Materials

The following supporting information can be downloaded at: https://www.mdpi.com/article/10.3390/nano1010000/s1.
