# Supplementary material for: Lossy Mode Resonance Sensors Based on Anisotropic Few-Layer Black Phosphorus
Source: Nanomaterials (Basel). 2024 Apr 23;14(9):736. doi: 10.3390/nano14090736 (PMC11085111; doi:10.3390/nano14090736)
Supplement: Supplementary file 1 [file nanomaterials-14-00736-s001.zip › nanomaterials-2939773-supplementary.pdf]

Lossy Mode Resonance Sensors Based on Anisotropic Few-Layer Black Phosphorus

Supplemental Material

1. Comparison of sensing parameters between "MgF2 − few layer black phosphorus (FLBP) −MgF2" LMR sensor and Au SPR sensor.

The reflectivity, sensitivity and FOM of BK7-50 nm Au SPR Kretschmann structure sensor has been analysed, shown in Fig. 1s. The refractive index of the sensing media changes from 1.38 to 1.3808 with 0.0002 interval. The Au SPR structure show much higher angular sensitivity nearly 200°/RIU comparing to 90°/RIU of BP LMR sensor. However, it is obvious that the reflectance curves of different sensing media are close together with low dispersion. Therefore, it shows low FOM of 34.6 /RIU comparing to 1.178×10^6 /RIU of BP LMR.

As a result, though the Au SPR sensor show higher sensitivity, its FOM is extremely low comparing to BP LMR sensor. This is the main advantage that distinguishes LMR sensors from SPR sensors.

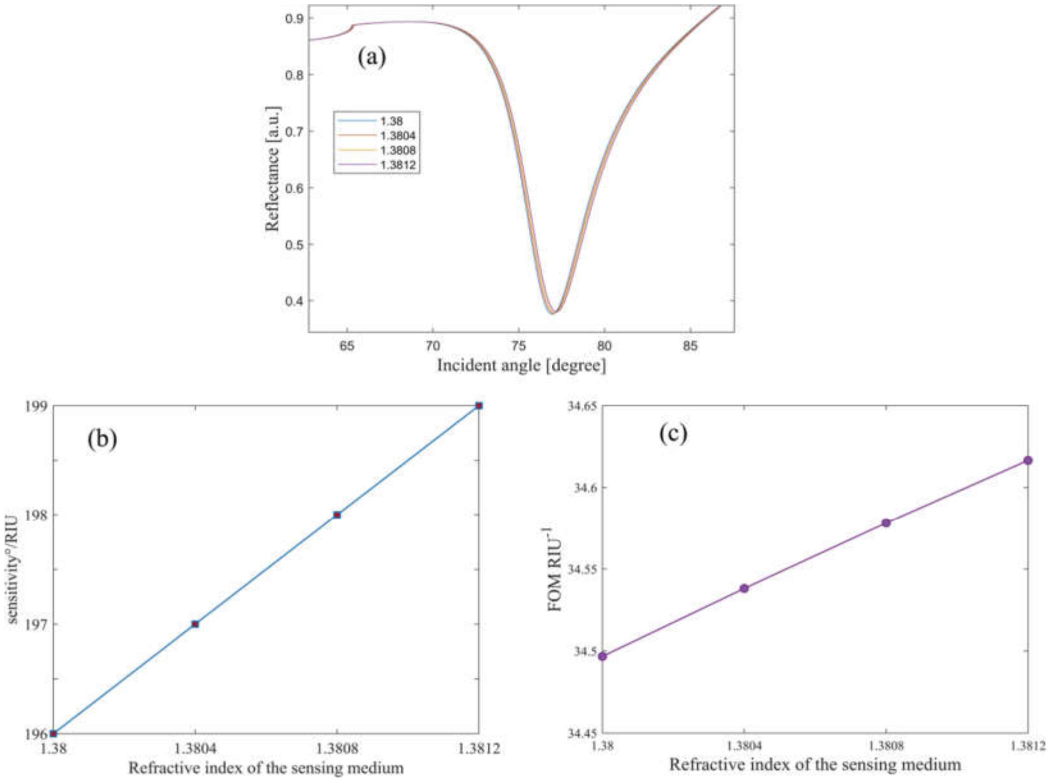

Figure S1. (a) Reflectivity, (b) sensitivity, and (c) FOM of the BK7- 50 nm Au SPR Kretschmann structure sensor.

2. The influence of the thickness of BP on sensing performance

1) Reflectivity of "MgF_2−FLBP−MgF_2" LMR sensors based on 1-7 layer BP

Fig.2s illustrate the reflectivity of "MgF_2−zz stacking BP−MgF_2" LMR sensors under TE wave condition. With increasing BP thickness, the resonance in decreasing. Therefore, we only analysed 3 layer condition. Fig.3s illustrate the reflectivity of "MgF_2−FLBP−MgF_2" LMR sensors under TM wave condition. The thickness of BP changes from 3 to 7 layers. The resonance reaches maximum with 5 layer BP.

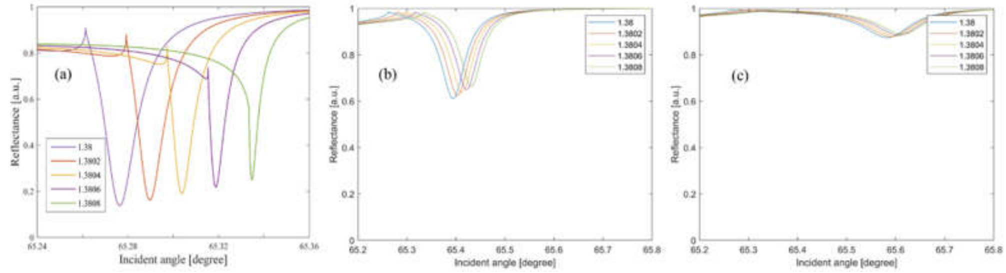

Figure S2. Reflectivity of LMR sensors based on (a) 1 layer BP, (b) 2 layer BP, and (c) 3 layer BP under TE incident light.

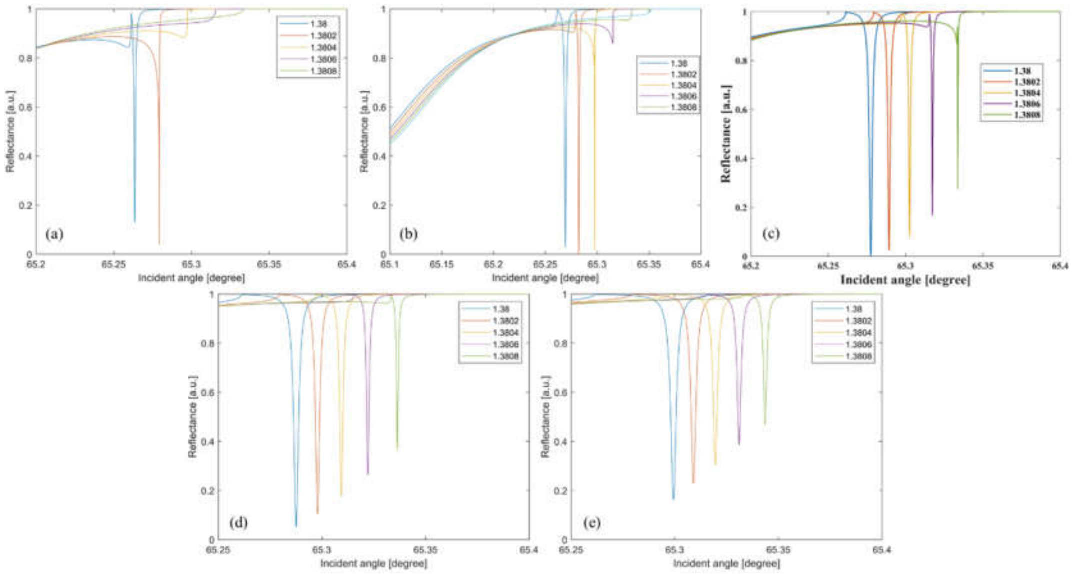

Figure S3. Reflectivity of LMR sensors based on (a) 3 layer BP, (b) 4 layer BP, (c) 5 layer BP, (d) 6 layer BP, and (e) 7 layer BP under TM incident light.

2) Sensitivity and FOM of LMR sensors based on 1-7 layer BP

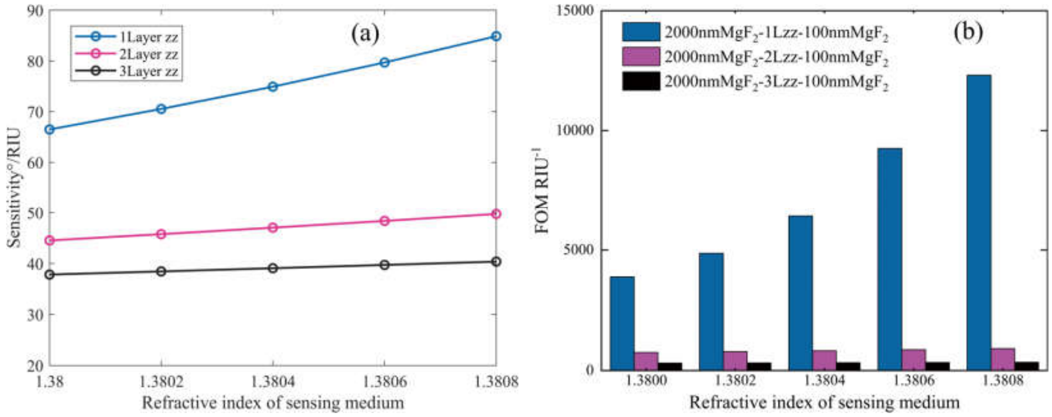

Figure S4. (a) Sensitivity and (b) FOM of LMR sensors with 1/2/3 layer BP under TE incident light.

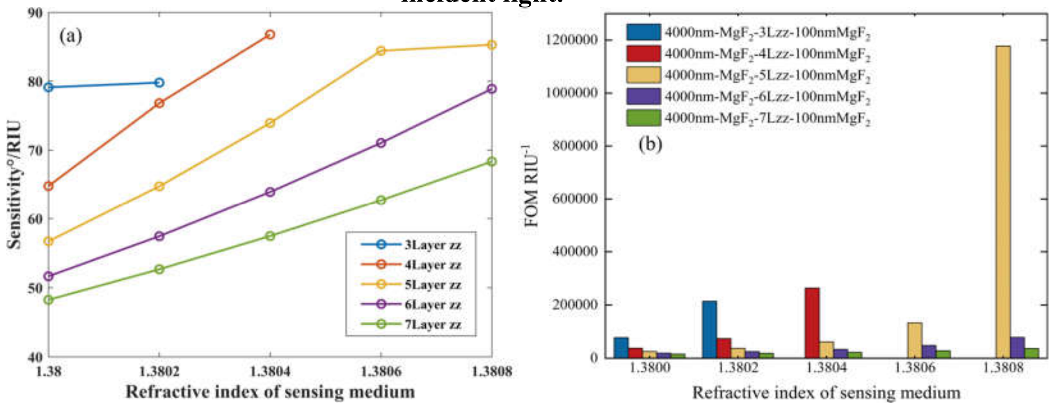

Figure S5. (a) Sensitivity and (b) FOM of LMR sensors with 3/4/5/6/7 layer BP under TM incident light.

3. **The influence of the thickness of the matching layer MgF_2 on sensing performance**

Fig. 6s shows the reflectivity of “MgF_2−5LBP−MgF_2” LMR sensors. The first MgF_2 layer is the matching layer. The other MgF_2 layer is the protective layer. Here, the thickness of the matching layer is analysed from 3000 nm to 5000 nm with 500 nm interval.

Therefore, the sensor could achieve higher sensing performance with 4000 nm MgF_2.

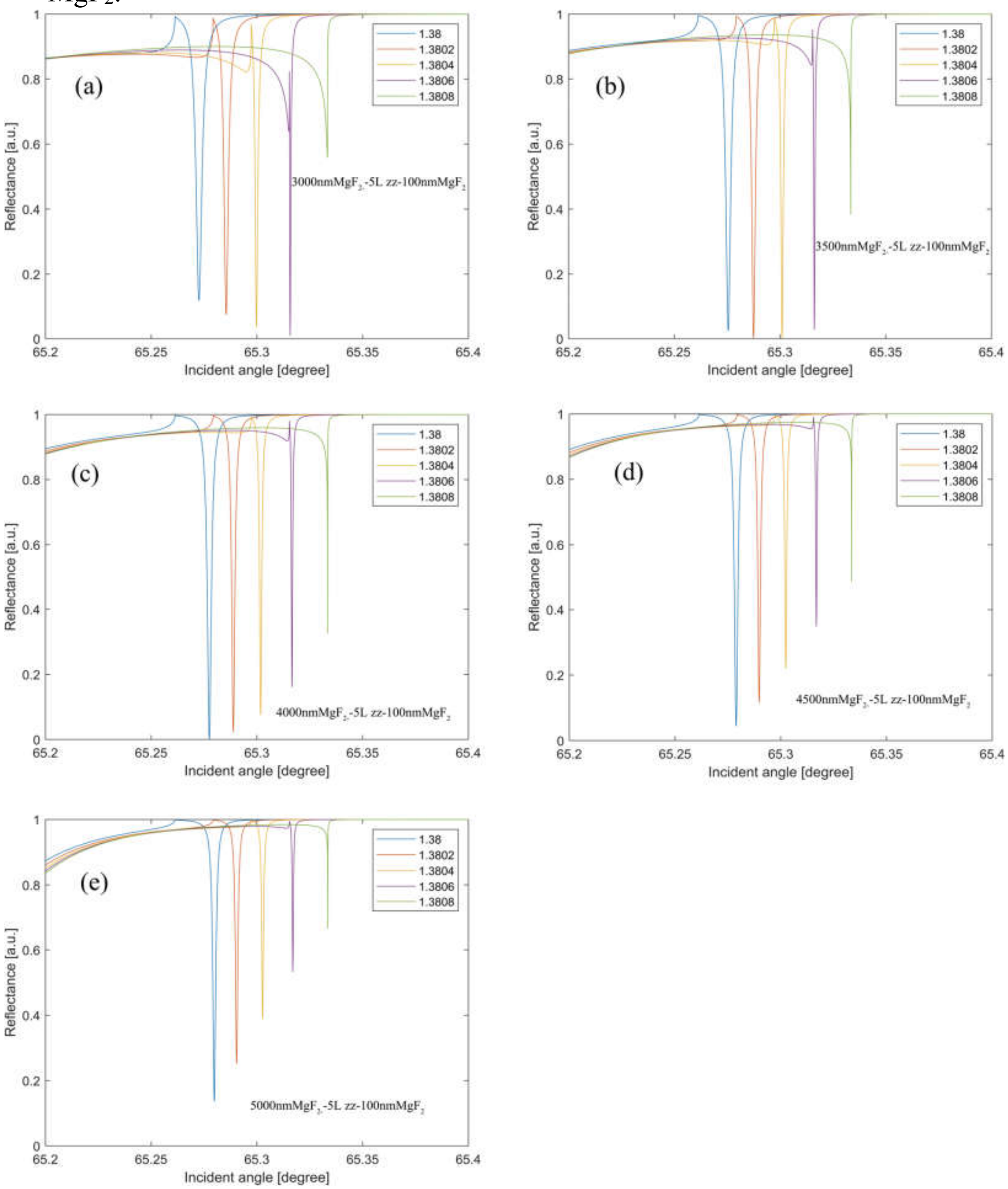

Figure S6. Reflectivity of LMR sensors with (a) 3000 nm, (b) 3500 nm, (c) 4000 nm, (d) 4500 nm, and (e) 5000 nm MgF_2 and 5 layer BP under TM incident light.

4. The influence of the stacking sequences of BP on sensing performance

We did a comparison of sensing performance of five-layer BP with 4 different stacking sequences, including 5zz, zz+ac+zz+ac+zz, ac+zz+ac+zz+ac, and 5ac. Their reflectivity, sensitivity, and FOM are shown below:

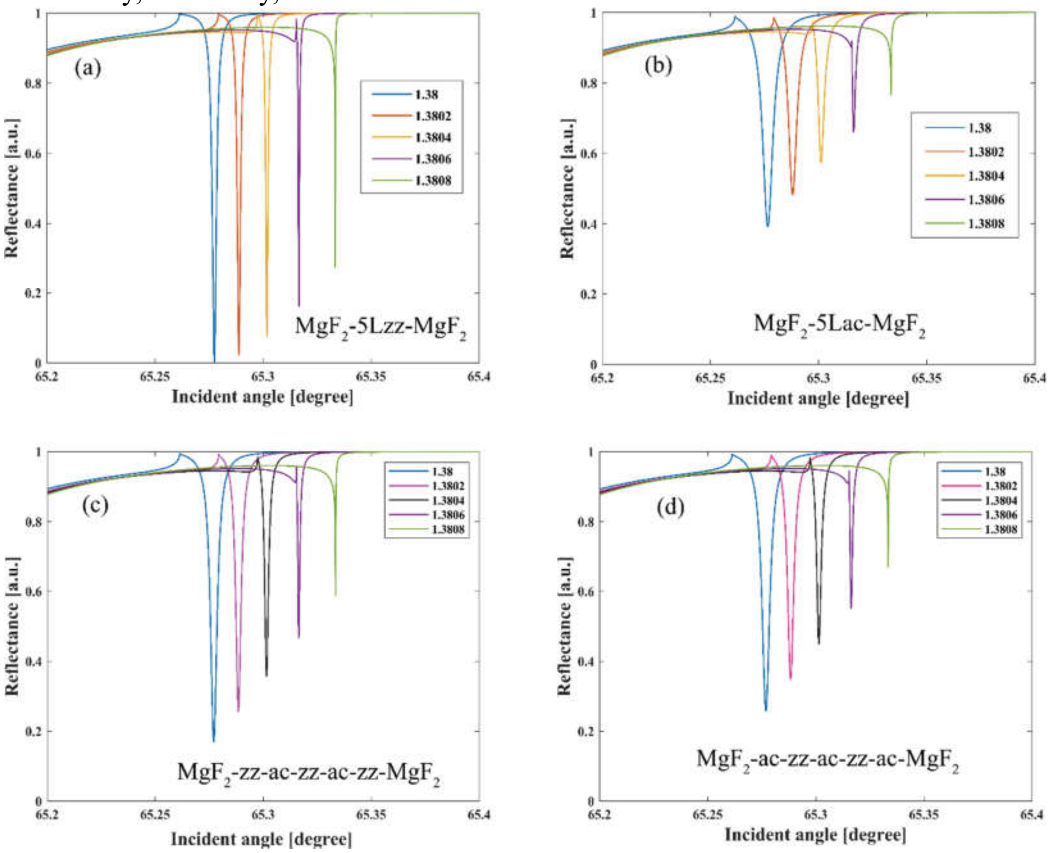

Figure S7. Reflectivity of "MgF2-5LBP-MgF2" LMR sensors under TM light with (a) 5zz stacking BP, (b) 5ac stacking BP, (c) zz-ac-zz-ac-zz stacking BP, and (d) ac-zz-ac-zz-ac stacking BP, respectively.

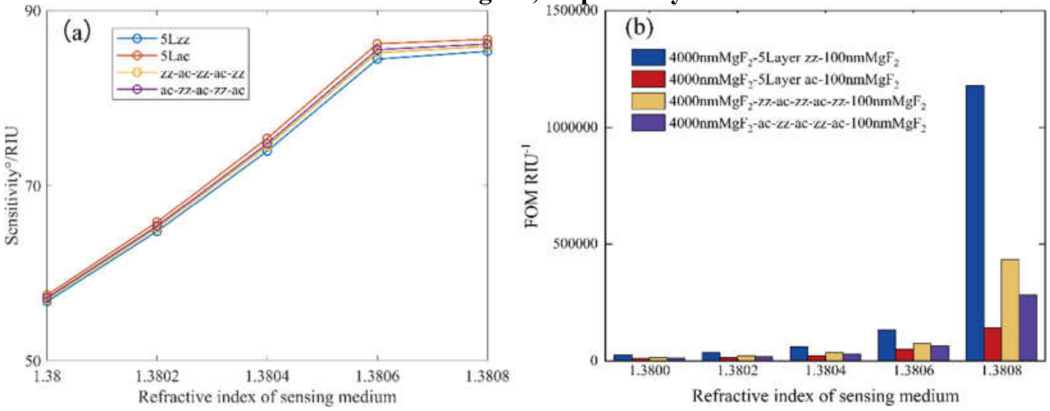

Figure S8. **(a) Sensitivity and (b) FOM of "MgF2-5LBP-MgF2" LMR sensors under TM light with different BP stacking sequences.**

It is obvious that 5L zz stacking BP could achieve stronger resonance and higher FOM, which confirms the significant impact of the stacking sequences of BP on sensing performance.

5. Analysis of BP-based LMR sensors in an oxygen isolated environment

We examined the MgF_2−FLBP LMR structure, which lacks the protective MgF_2 layer. Under TM polarization, the optimal thicknesses for the matching and lossy layers are d_1=4000 nm and d_2 =2.65 nm (5-layer BP), respectively. Fig. 9s (a) to (d) illustrates the reflectance, sensitivity, and FOM of the MgF_2−5LBP sensors with zz and ac BP. Analogous to the MgF_2−5LBP−MgF_2 structure, the maximum FOM is achieved when n_s = 1.3808, reaching up to 3.192×10^5 RIU^{-1}. Therefore, a protective MgF_2 layer is also able to enhance LMR, which will be confirmed by the following electric field analysis. The electric field of the 4000 nm MgF_2−5LBP configuration, shown in Fig. 9s (e) and (f), is marginally lower than that of the 4000 nm MgF_2−5LBP−100 nm MgF_2 configuration, aligning with the sensitivity and FOM analyses.

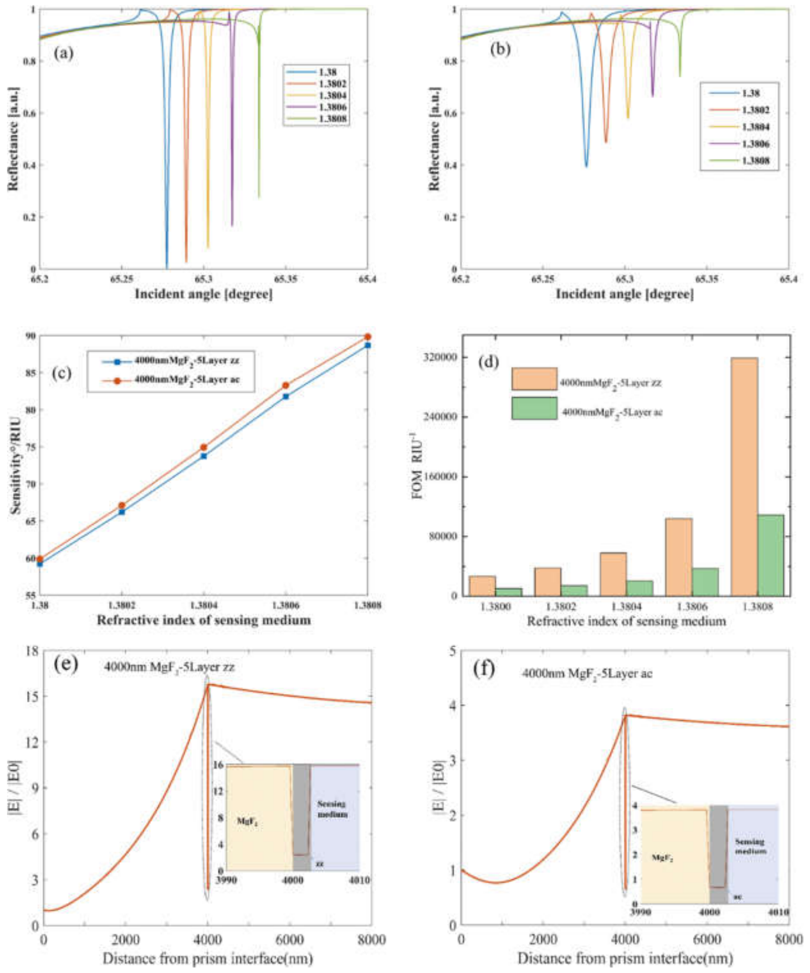

Figure S9. Comparison of the LMR sensing performance of "4000 nm MgF2−5L zz BP" structure and "4000 nm MgF2−5L ac BP" structure under TM polarized light: a) and b) The reflectance of structures with 5L zz BP and 5L ac BP respectively; c) The resonant angular sensitivity and d) FOM of the two structures; the electric field distribution of e) 4000 nm MgF_2−5L zz BP and f) 4000 nm MgF_2−5L ac BP. The embedded diagrams of (e) and (f) are the magnified electric field distribution around the FLBP interfaces.
